# The Prevalence of Sarcopenia and Its Impact on Clinical Outcomes in Lumbar Degenerative Spine Disease—A Systematic Review and Meta-Analysis

**DOI:** 10.3390/jcm10040773

**Published:** 2021-02-15

**Authors:** Wei-Ting Wu, Tsung-Min Lee, Der-Sheng Han, Ke-Vin Chang

**Affiliations:** 1 Department of Physical Medicine and Rehabilitation, National Taiwan University Hospital, Bei-Hu Branch, Taipei 108, Taiwan; wwtaustin@yahoo.com.tw (W.-T.W.); dshan1121@yahoo.com (D.-S.H.); 2 Department of Physical Medicine and Rehabilitation, National Taiwan University College of Medicine, Taipei 100, Taiwan; braden2011@hotmail.com.tw

**Keywords:** sarcopenia, frailty, lumbar spondylosis, spinal stenosis, aging

## Abstract

The association of sarcopenia with poor clinical outcomes has been identified in various medical conditions, although there is a lack of quantitative analysis to validate the influence of sarcopenia on patients with lumbar degenerative spine disease (LDSD) from the available literature. Therefore, this systematic review and meta-analysis aimed to summarize the prevalence of sarcopenia in patients with LDSD and examine its impact on clinical outcomes. The electronic databases (PubMed and Embase) were systematically searched from inception through December 2020 for clinical studies investigating the association of sarcopenia with clinical outcomes in patients with LDSD. A random-effects model meta-analysis was carried out for data synthesis. This meta-analysis included 14 studies, comprising 1953 participants. The overall prevalence of sarcopenia among patients with LDSD was 24.8% (95% confidence interval [CI], 17.3%–34.3%). The relative risk of sarcopenia was not significantly increased in patients with LDSD compared with controls (risk ratio, 1.605; 95% CI, 0.321–8.022). The patients with sarcopenia did not experience an increase in low back and leg pain. However, lower quality of life (SMD, −0.627; 95% CI, −0.844–−0.410) were identified postoperatively. Sarcopenia did not lead to an elevated rate of complications after lumbar surgeries. Sarcopenia accounts for approximately one-quarter of the population with LDSD. The clinical manifestations are less influenced by sarcopenia, whereas sarcopenia is associated with poorer quality of life after lumbar surgeries. The current evidence is still insufficient to support sarcopenia as a predictor of postoperative complications.

## 1. Introduction

Low back pain is one of the most common musculoskeletal disorders and its prevalence ranges between 1.4% and 15.6% in the general population [1]. In older adults, the prevalence of low back pain can increase up to 36%–70% [2]; additionally, lumbar degenerative spine disease (LDSD) account for the majority of radiographic pathologies [3]. LDSD is characterized by the degeneration of intervertebral discs, overgrowth of osteophytes and facet hypertrophy. It has been observed that some patients develop spinal stenosis and narrowing of the spinal canal leads to severe low back and leg pain due to the compression of the spinal cord and nerves. A systematic review indicated that patients with LDSD had the lowest quality of life among all the disease states examined, including prostate cancer, type II diabetes, chronic obstructive pulmonary disease, inflammatory bowel disease, renal failure, rheumatoid arthritis, congestive heart failure, knee osteoarthritis, hip osteoarthritis and peripheral vascular disease [4]. The treatments for LDSD include medications, physical therapy and minimally invasive procedures (such as injection and radiofrequency ablations) [5]. Surgeries, such as laminectomy and lumbar interbody fusion, may be suggested for patients who are resistant to the aforementioned managements; however, complications may occasionally develop in some postoperative cases, leading to more disability [6,7].

Sarcopenia is a skeletal muscle disorder defined as a decline in muscle mass and strength. The association of sarcopenia with poor clinical outcomes has been identified in several medical conditions, such as chronic obstructive pulmonary disease [8], coronary heart disease [9], chronic kidney disease [10] and various types of cancer [11,12]. The mechanism behind poor prognoses in sarcopenic patients is multifactorial, encompassing disrupted muscle protein homeostasis, a decrease in the physical reserve to overcome a stress event and enhanced systemic inflammation [13]. The relationship between sarcopenia and LDSD has been investigated recently. In 2018, a systematic review encompassing 11 articles investigated the role of sarcopenia and frailty in adults undergoing spine surgeries [14]. The authors found an inconsistent relationship between sarcopenia and postoperative outcomes due to a lack of consistent criteria in the diagnosis of sarcopenia. In 2020, a narrative review addressed the impact of sarcopenia on LDSD, revealing that the existence of sarcopenia in patients preoperatively might result in deteriorated postsurgical outcomes [15]. To date, there is a lack of quantitative analyses to investigate the influence of sarcopenia on patients with LDSD from the available literature. Therefore, the aim of the present systematic review and meta-analysis was threefold: (1) to survey the prevalence of sarcopenia in patients with LDSD; (2) to explore whether patients with LDSD were at a higher risk of sarcopenia than the controls and (3) to investigate whether sarcopenia led to worse clinical outcomes in the population with LDSD.

## 2. Methods

### 2.1. Data Sources and Search Strategy

This systematic review and meta-analysis was based on a pre-planned protocol constructed in accordance with the standard Preferred Reporting Items for Systematic Reviews and Meta-Analysis (PRISMA) [16] and was prospectively registered on inplasy.com (INPLASY2020120123). A systemic literature search was conducted in PubMed (US National Library of Medicine) and Embase (Wolters Kluwer Ovid) for observational and cohort studies investigating sarcopenia in patients with LDSD. The combinations of the following keywords were employed for the search, including sarcopenia, frailty, low back pain, lumbar spinal stenosis, lumbar spondylosis thesis and lumbar degenerative disc disease (Appendix A). The electronic databanks were searched from their earliest records to December 2020. Furthermore, manual retrieval was performed from relevant narrative and systemic reviews.

### 2.2. Inclusion and Exclusion Criteria

All observational and cohort studies were included if they conformed to the following criteria: (1) original research investigating the association of sarcopenia with clinical outcomes in patients with LDSD; (2) inclusion of middle-aged or older adults (age ≥50 years) and (3) with a clearly defined algorithm to differentiate participants with and without sarcopenia. The exclusion criteria were as follows: (1) case reports, case series, reviews, study protocols, editorials or commentaries; (2) lack of definition for sarcopenia; and (3) enrollment of patients with spinal pathologies other than the lumbar region.

### 2.3. Data Extraction

Following the search of pertinent literature from the aforementioned databases, two authors began to scrutinize relevant abstracts independently. If there was any disagreement between the two reviewers regarding the eligibility of the reviewed articles, a decision was made by discussions or opinions of the corresponding author. The full texts of the eligible articles were subsequently retrieved and the data were extracted using a standardized form in Microsoft Excel. The excerpted information consisted of the name of the first author, year of publication, study type, participants’ characteristics, diagnosis of lumbar spine diseases, the definition of sarcopenia, measurements of muscle mass and function, clinical outcomes before and after surgeries, surgical procedures and major postoperative complications.

### 2.4. Study Quality Assessment

The modified Newcastle Ottawa scale for non-randomized trials was used to assess the quality of the studies included in the meta-analysis [17]. The quality assessment was conducted by both aforementioned reviewers individually, while the result of the evaluation was achieved by discussion or decision of the corresponding author. Several aspects were appraised, including the representativeness of the participants, appropriateness of the sample size, ascertainment of exposure, documents of non-respondents, comparability of different groups, assessment of outcomes and sufficient follow-up duration.

### 2.5. Statistical Analysis

The primary outcome was the prevalence of sarcopenia in patients with LDSD, which was assumed to be a binomial distribution. The variance of the prevalence value was calculated as follows: (1-prevalence) * prevalence/number of the population size. Comparisons of sarcopenia between patients with LDSD and controls or clinical outcomes between patients with and without sarcopenia were quantified using the risk ratio (RR). A subgroup analysis was performed based on the differences in the diagnostic criteria for sarcopenia. The standardized mean difference (SMD) was used to compare the continuous variables, derived from the differences between the means divided by the pooled standard deviations. Data pooling was achieved by using the random effect model [18], considering differences in the patient population across the included studies. The I^2^ statistic was employed to evaluate the heterogeneity of the enrolled studies and I^2^ > 50% was regarded as substantial heterogeneity [19]. The potential existence of publication bias was determined by the Egger test and visual inspection of the distributions of the effect size on the funnel plot [20]. A two-sided P value <0.05 was considered statistically significant and all analyses were implemented using Comprehensive Meta-analysis Software v 3 (Biostat, Englewood, NJ, USA).

## 3. Results

### 3.1. Literature Search

**Figure 1:**
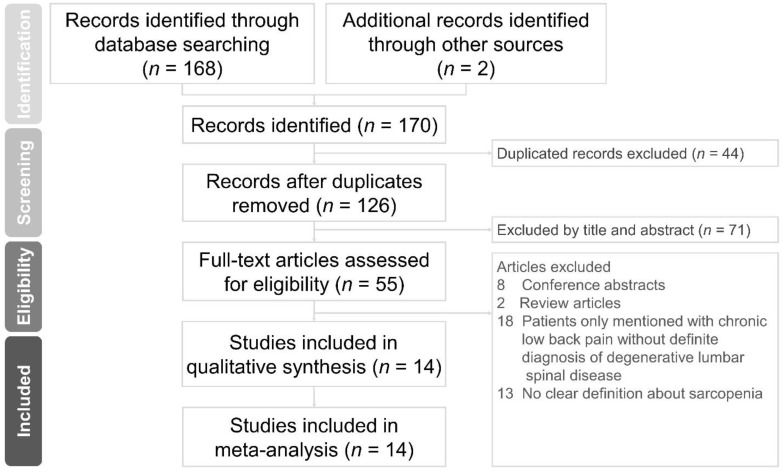
Preferred Reporting Items for Systematic Reviews and Meta-Analyses (PRISMA) flow diagram for the study selection process.

A total of 170 articles were accessed from all databases. After eliminating duplicates, 126 articles were left, 55 of which were pertinent to our topic after surveying their titles and abstracts. After screening the full text of the 55 articles, 14 met the inclusion criteria [21,22,23,24,25,26,27,28,29,30,31,32,33,34] and were further enrolled in the meta-analysis, comprising 1953 participants. The flow diagram of the literature search is shown in Figure 1.

### 3.2. Study Characteristics

Of the fourteen included studies, seven used a cross-sectional design [24,26,27,28,29,32,33] and seven used a cohort design [21,22,23,25,30,31,34] (Table 1). Their average age ranged between 63.3 and 76.9 years and women accounted for 50.9% of the overall participants. Sarcopenia was defined by using the consensus of the Asian Working Group for Sarcopenia (AWGS) in nine studies [25,26,27,28,29,30,31,32,33], using the criteria specific to the skeletal muscle mass index of the Japanese population in two studies [23,24] and using only the psoas muscle cross-sectional area derived from computed tomography or magnetic resonance imaging in three studies [21,22,34] (Table 2). Regarding the measurement of muscle mass, 3 studies employed dual-energy x-ray absorptiometry [23,24,25], 5 studies employed bioelectrical impedance analysis [28,29,30,32,33], 3 studies employed computed tomography [21,22,26] and one study employed magnetic resonance imaging [34]. The skeletal muscle volume was not measured in two studies [27,31]. In terms of muscle function, the grip strength measured by hand dynamometers was reported in eight studies [26,27,28,29,30,31,32,33]. Regarding the diagnosis in the patient group, lumbar spinal stenosis accounted for the majority of lumbar spine pathologies. A total of six studies evaluated the clinical outcomes before and after lumbar spine surgery [27,28,30,31,32,33] (Table 1).

#### 3.2.1. Quality Assessment of the Included Studies

The results of the quality assessment are presented in Table 3. The domain that failed the most was “comparability of different groups.” The majority of the included studies did not have a comparative group without LDSD. The second most failed item was “enough follow-up durations.” The cross-sectional design was employed in the majority of the enrolled articles without the following up of patients’ postoperative outcomes.

#### 3.2.2. Prevalence of Sarcopenia in LDSD

**Figure 2:**
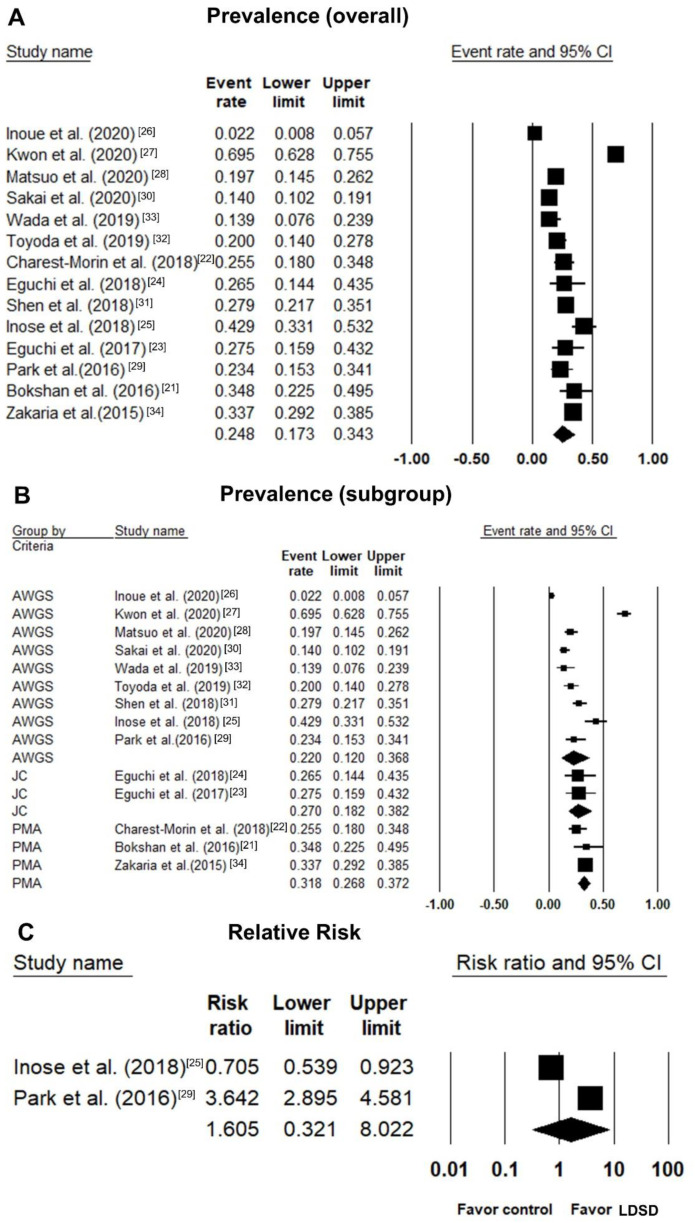
Forest plot of the overall prevalence of sarcopenia in patients with lumbar degenerative spine disease (**A**); the subgroup analysis of the prevalence of sarcopenia based on the different diagnostic criteria (**B**); the relative risk of sarcopenia in the group with lumbar degenerative spine vs. controls (**C**).;AWGS: the consensus of Asian Working Group for Sarcopenia; JC: criteria specific for the skeletal muscle mass index of the Japanese population; PMA: psoas muscle cross-sectional area; CI: confidence interval; LDSD: lumbar degenerative spine disease.

**Figure 3:**
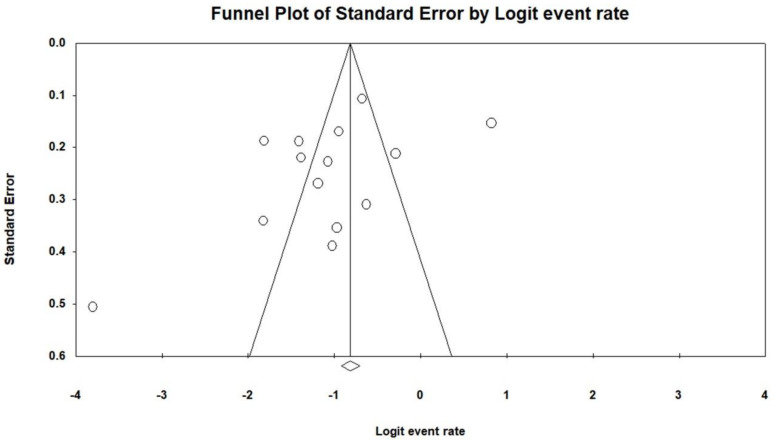
Funnel plot for the log-transformed point estimates of the prevalence among the included studies.

The overall prevalence of sarcopenia among patients with LDSD was 24.8% (95% confidence interval [CI]: 17.3%–34.3%; I^2^ statistics: 93.9%) (Figure 2A). A subgroup analysis was performed based on the definition of sarcopenia. The pooled prevalence was 22.0% (95% CI: 12.0%–36.8%; I^2^ statistics: 96.2%) in the studies using AWGS, 27.0% (95% CI: 18.2%–38.2%; I^2^ statistics: <0.01%) in the studies using the criteria of the skeletal muscle mass index specific for the Japanese population and 31.8% (95% CI: 26.8%–37.2%; I^2^ statistics: 23.4%) in the studies using only the psoas muscle cross-sectional area (Figure 2B). Egger’s regression test revealed no evidence of significant publication bias (p = 0.11) (Figure 3).

#### 3.2.3. Relative Risk of Sarcopenia in LDSD vs. Controls

Only two studies [25,29] enrolled participants without spine conditions as controls, which allowed the estimation of the relative risk of sarcopenia in participants with vs. without LDSD (RR: 1.605, 95% CI: 0.321–8.022; I^2^ statistics: 98.79%).

### 3.3. Clinical Outcomes

#### 3.3.1. Low Back Pain

**Figure 4:**
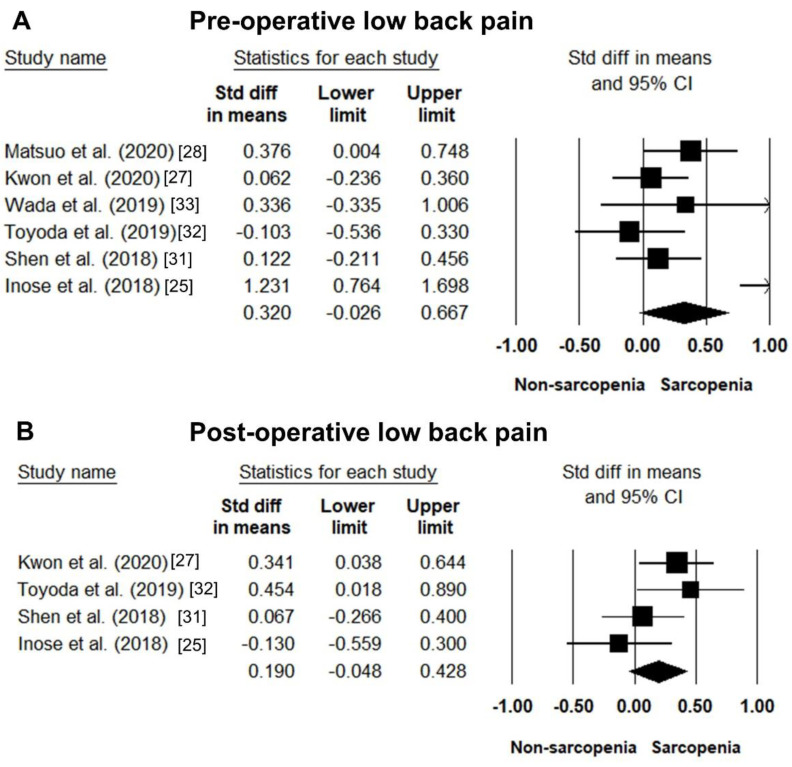
Forest plot of the standardized mean differences of the pre-operative (**A**) and post-operative (**B**) low back pain between patients with and those without sarcopenia. Std diff: standardized difference.

The data of preoperative low back pain were available in six studies [25,27,28,31,32,33]. The pooled SMD of sarcopenic vs. non-sarcopenic participants was 0.320 (95% CI: −0.026–0.667; I^2^ statistics: 77.3%) (Figure 4A). Likewise, four studies reported data on low back pain after lumbar surgeries [25,27,31,32]. The pooled SMD was 0.190 (95% CI: −0.048–0.428; I^2^ statistics: 40.0%) (Figure 4B).

#### 3.3.2. Leg Pain

**Figure 5:**
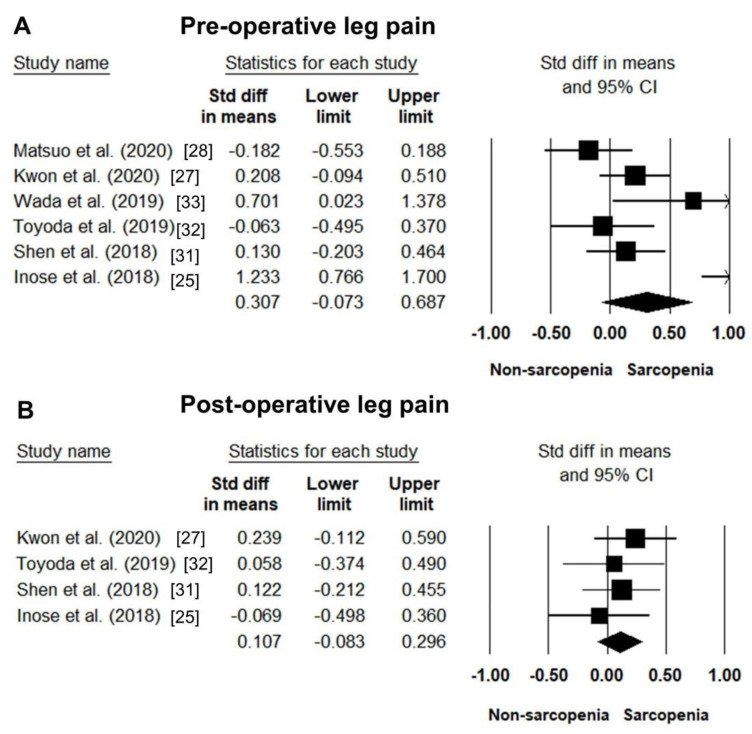
Forest plot of the standardized mean differences of the pre-operative (**A**) and post-operative (**B**) leg pain between patients with and those without sarcopenia.

Preoperative leg pain data could be obtained from six studies [25,27,28,31,32,33]. The pooled SMD of sarcopenic vs. non-sarcopenic participants was 0.307 (95% CI: −0.073–0.687; I^2^ statistics: 81.0%) (Figure 5A). Furthermore, the evaluations of postoperative leg pain were available in four studies [25,27,31,32], with a pooled SMD of 0.107 (95% CI: −0.083–0.296; I^2^ statistics: < 0.01%) regarding the comparison between sarcopenic and non-sarcopenic participants (Figure 5B)

#### 3.3.3. EuroQol-5D (EQ-5D)

**Figure 6:**
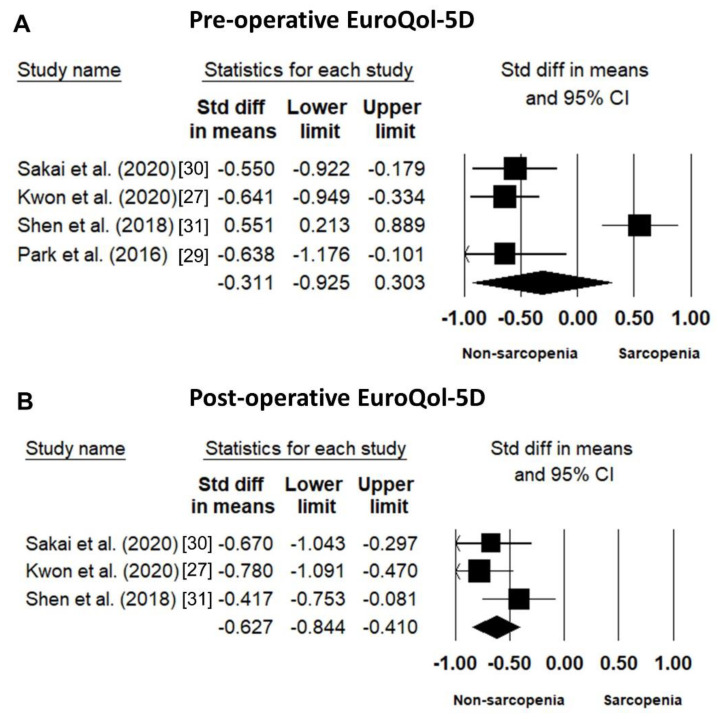
Forest plot of the standardized mean differences of the pre-operative (**A**) and post-operative (**B**) EuroQol-5D (EQ-5D) between patients with and those without sarcopenia.

The EQ-5D, an instrument for assessing the quality of life, was recorded preoperatively in four studies [27,29,30,31]. The pooled SMD of sarcopenic vs. non-sarcopenic participants was −0.311 (95% CI: −0.925–0.303; I^2^ statistics: 90.69%). In addition, three studies had a postoperative evaluation of EQ-5D [27,30,31] (Figure 6A). The pooled SMD of sarcopenic vs. non-sarcopenic participants was −0.627 (95% CI: −0.844–−0.410; I^2^ statistics: 19.38%), indicating the significantly worse quality of life in patients with sarcopenia than in those without sarcopenia (Figure 6B).

#### 3.3.4. Post-Operative Complication

**Figure 7:**
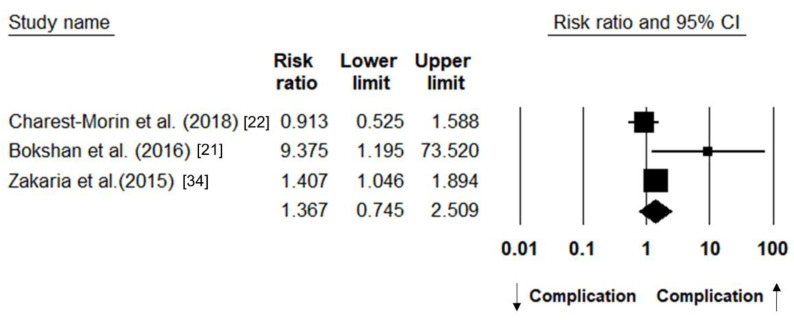
Forest plot of the relative risk of post-operative complications between patients with and those without sarcopenia; ↓ indicates a decrease in the risk, whereas ↑ indicates an increase in the risk.

The number of patients with postoperative complications was detailed in three studies [21,22,34]. The pooled RR of sarcopenic vs. non-sarcopenic participants was 1.367 (95% CI: 0.745–2.509; I^2^ statistics: 62.69%) (Figure 7).

## 4. Discussion

The primary aim of this meta-analysis was to compile the currently available references to investigate the relationship between sarcopenia and LDSD in middle-aged and older adults. Our pooled results showed that the prevalence of sarcopenia in patients with LDSD was approximately 25% and was not higher than that in the controls without lumbar spine pathology. Regarding clinical outcomes, sarcopenia did not lead to significant differences in clinical manifestations. However, poorer quality of life was presented in post-operative patients with sarcopenia. Finally, there was no evidence to prove that the patients with sarcopenia had a higher rate of complications after lumbar spine surgeries.

Our study revealed that patients with sarcopenia accounted for one-quarter of the population with lumbar degenerative spine disease. A recent meta-analysis pointed out that the prevalence of sarcopenia was 11% (95% CI: 8–13%) in men and 9% (95% CI: 7–11%) among community-dwelling residents [35]. For individuals who were hospitalized or lived in nursing homes, the prevalence of sarcopenia increased to between 23% and 51% [35]. Another meta-analysis demonstrated that sarcopenia was highly prevalent in adults with cardiovascular diseases, dementia, diabetes mellitus and respiratory disease, with a pooled prevalence of 31.4%, 26.4%, 31.1% and 26.8%, respectively [36]. Compared with the data obtained from the aforementioned meta-analyses, our results indicated that the prevalence of sarcopenia in patients with LDSD appeared higher than in community-dwelling individuals and was similar to the participants from residential facilities or with comorbidities. We speculated that patients with LDSD were characterized by old age and physical inactivity due to pain and weakness, which were also the risks for the decline in muscle mass and function.

Compared with the control group, there was no strong evidence to prove a higher risk of sarcopenia in patients with LDSD. Two possible reasons might account for this finding. First, only two studies included participants without lumbar pathology as the control. Although the point estimate (risk ratio = 1.605) revealed a likely higher prevalence of sarcopenia in the patient group, the sample size was insufficient to achieve appropriate statistical power. Second, the development of sarcopenia is multi-factorial, comprising malnutrition, physical inactivity and chronic inflammation [13,37]. The matched controls were highly likely to share certain risk factors with patients with LDSD, such as sedentary lifestyle and age-related comorbidity, which led to no significant differences in the prevalence of sarcopenia between the population with and without LDSD.

Our results revealed that sarcopenia did not lead to worse clinical manifestations. Theoretically, the patients with sarcopenia also suffer from loss of the axial lean mass and weakness of core muscles, which affects their trunk stability during movement. Their facet joints and adjacent spinal nerves are vulnerable to hypermobility as a consequence of abdominal and paraspinal muscle atrophy. However, the patients’ presentation and prognosis are influenced by several factors, such as spine alignment, number of involved vertebrae and concomitant neurological conditions (such as peripheral neuropathy) [5]. Therefore, sarcopenia is unlikely to act as an exclusive moderator of the intensity of low back and leg pain in the population with LDSD.

Our results revealed that patients with sarcopenia had lower quality of life than non-sarcopenic participants after lumbar spine surgeries. The SMD for the quality of life was -0.627, denoting a medium to large effect size [38]. Sarcopenia has been reported to be associated with adverse post-surgical outcomes in many medication conditions. In 2020, a meta-analysis reported an elevated risk of complications and readmissions in patients with sarcopenia after any type of gastrointestinal surgery [39]. Similarly, another meta-analysis pointed out that sarcopenia independently predicted shorter survival and increased mortality among patients undergoing urologic oncology surgeries [40]. As lumbar spine surgeries inevitably cause various grades of the destruction of the paraspinal muscles, the dynamic stability over the axial skeleton would be further compromised, leading to more impairment of daily activities. Therefore, our meta-analysis revealed that although the differences in clinical presentations between patients with and without sarcopenia were not distinct, the operation-related physical and psychological stress would potentiate the influence of sarcopenia and lead to worse post-surgical quality of life measurements. Furthermore, sarcopenia decreases the regenerative capacity of skeletal muscles following satellite cell loss and dysfunction. A decline in satellite cell function and numbers is related to age-dependent muscle fibrosis, further deteriorating the recovery potential of sarcopenic muscles subsequent to injury [41]. We speculated that replacement of normal muscles with scar tissues (in addition to atrophy of muscle fibers) in sarcopenic patients also played a role in poorer quality of life measurements after destruction of muscle tissue after lumbar surgeries.

In our meta-analysis, we did not identify a higher complication rate after lumbar spine surgery in patients with sarcopenia than in those without sarcopenia. We speculated that there might be three reasons for the aforementioned finding. First, the types and extents of surgeries varied from individual to individual, which was directly associated with post-surgical outcomes. Second, the patterns and severity of complications differed from study to study and the heterogeneity was likely to mitigate the impact of sarcopenia on the development of postoperative adverse events. Therefore, additional cohort studies are needed in the future to validate whether sarcopenia is an independent predictor of postoperative complications.

Several limitations of this study need to be acknowledged. First, the diagnostic criteria for sarcopenia varied among the included studies. Even in the studies that used the consensus of AWGS, not all of them measured both body compositions and grip strength. The aforementioned variations inevitably led to significant heterogeneity in our results. Second, only parts of the enrolled studies reported the needed clinical outcomes, which diminished the power of the pooled effect sizes and possibly increased the risk of selective reporting bias. Third, it would be of clinical interest to know which kind of degenerative spine pathology would affect the muscle mass and function most and leaded to subsequent sarcopenia. However, like what we presented in Table 1, mixed types of spine degenerative pathologies (e.g., lumbar spine stenosis, degenerative lumbar spondylolisthesis, degenerative lumbar scoliosis, lumbar compression fracture and lumbar kyphosis) were included in the majority of the retrieved studies. Therefore, the subgroup analysis could not be performed based on the differences in spinal pathologies of individual studies in our meta-analysis. Fourth, physical therapy is important for restoration of muscle mass and function and would be beneficial for patients with sarcopenia. However, whether the patients received physical therapy was rarely reported in the included studies. The majority of the retrieved studies focused on the types of spine surgeries and post-surgical outcomes. Therefore, it is hard to know the actual benefits of physical therapy for prevention and treatment of sarcopenia in the current patient population. More prospective trials are definitely needed to investigate the advantages of physical therapy for sarcopenia in patients with LDSD.

## 5. Conclusions

Sarcopenia is prevalent in middle-aged and old adults with LDSD, accounting for approximately one-quarter of the patient population. The prevalence of sarcopenia is not higher in patients with LDSD than in the controls.The clinical manifestations are less influenced by sarcopenia, whereas sarcopenia is likely to be associated with poorer quality of life after lumbar surgeries. The current evidence is still insufficient to support sarcopenia as a predictor of postoperative complications.

## Figures and Tables

**Table 1:** Characteristics of the Included Studies.

Author, Year	Study Design	Patient Characteristic	Type of Lumbar Spine Surgery	Outcome	*n* Total	Age (Year)	Sex Ratio: M/F	Data Collection Period	Country
Inoue et al. (2020) [26]	Cross-sectional	Patients with LSS	Not specified	Intermittent claudication(m), PMI, BMI, Comorbidity	183	70.5 ± 8.6	128/55	2015/06–2018/03	Japan
Kwon et al. (2020) [27]	Cross-sectional	Patients with LSS w or w/o spondylolisthesis	Decompression surgery, TLIF	ODI, EQ-5D, VAS (back/leg pain), AST, SMT, STS, TUGT	200	69.5 ± 6.5 (sarcopenia, F) 69.9 ± 6.5 (sarcopenia, M) 64.5 ± 7.1 (non-sarcopenic, F) 65.4 ± 9.7 (non-sarcopenic, M)	74/126	2014/04–2016/04	Korea
Matsuo et al. (2020) [28]	Cross-sectional	Patients with degenerative LSS	Nil	VAS (back/leg pain and numbness), SF36, JOABPEQ, BMD (lumbar, femoral), Spinal alignment	178	79.0 ± 1.2 (sarcopenic) 72.6 ± 0.6 (non-sarcopenic)	77/101	2017/09–2018/08	Japan
Sakai et al. (2020) [30]	Retrospective cohort	Patients with LSS w or w/o spondylolisthesis	Decompression surgery, PLIF	RDQ, SF36 PCS, EQ-5D, BMI, BMD, Spinal alignment	235	73.2 + 5.8	135/100	2014/04–2017/03	Japan
Wada et al. (2019) [33]	Cross-sectional	Patients with LSS	Nil	VAS (back/leg pain), JOA score (lower back dysfunction), PCS, HADS, FABQ	72	70.4 ± 6.9	38/34	2015/10–2018/04	Japan
Toyoda et al. (2019) [32]	Cross-sectional	Patients with LSS, degenerative lumbar spondylolisthesis, degenerative lumbar scoliosis	Minimally invasive lumbar decompression surgery	JOA score, VAS (back/leg pain and numbness)	130	76.9 ± 6.4	70/60	2015/10–2016/07	Japan
Charest-Morin et al. (2018) [22]	Retrospective cohort	Patients with LSS w or w/o spondylolisthesis, disc herniation	Decompression or fusion surgery	ASA, surgical factors, mFI, SSII, major complications, LOS	102	72 (IQR:68-78)	51/51	2009/01/01–2013/13/31	Canada
Eguchi et al. (2018) [24]	Retrospective cohort	Patients with LSS	Laminectomy, TLIF, OLIF	JOA score, RDQ, BMD, spinal alignment	34	74.4	Nil	2014/04–2016/10	Japan
Shen et al. (2018) [31]	Retrospective cohort	Patient with LSS w or w/o spondylolisthesis	Decompressive surgery w or w/o fusion (PLIF, TLIF)	PRO, ODI, EQ-5D, VAS (back/leg pain)	170	72.3 ± 6.6 (sarcopenic) 68.1± 9.2 (non-sarcopenic)	14/34 (sarcopenic) 63/61(non-sarcopenic)	2016/10–2017/06	Korea
Inose et al. (2018) [25]	Retrospective cohort	Patients with LSS, lumbar compression fracture, lumbar kyphosis	Wide fenestration, PLIF, vertebral column resection, Pedicle subtraction osteotomies	JOA score, VAS (back/leg and numbness)	91	74.8 ± 0.9 (sarcopenic) 73.0 ± 1.0 (non-sarcopenic)	17/20 (sarcopenic) 16/32 (non-sarcopenic)	2014/1/1–2015/12/31	Japan
Eguchi et al. (2017) [23]	Cross-sectional	Patients with LSS, lumbar scoliosis,	Laminectomies, corrective surgery	BMD, lean mass in body parts, JOA, RDQ Spinal alignment	40	74.0 ± 1.0	0/40	nil	Japan
Park et al.(2016) [29]	Cross-sectional	Patients with LSS	Nil	EQ-5D, BMI, STS, TUG, ODI, EQ5D	77	67.88 ± 6.91	18/59	2014/08–2014/11	Korea
Bokshan et al. (2016) [21]	Retrospective cohort	Patients with LSS, scoliosis, epidural abscess, discitis, acute fracture	Decompression w or w/o fusion	CCI, MSII, SII, postoperative complications, LOS, disposition at discharge	46	76.4 ± 8.8 (sarcopenic) 69.9 ± 10.95 (non-sarcopenic)	22/24	2003–2015/09	U.S.
Zakaria et al.(2015) [34]	Retrospective cohort	Patients receiving lumbar surgery	Laminectomy, OLIF, PLIF, MIS	LOS, disposition at discharge, any 90 day postoperative complications	395	63.30 ± 12.48	192/203	2013–2014	U.S.

LSS: lumbar spinal stenosis; PMI: psoas muscle index; BMI: body mass index; w: with; w/o: without; TLIF: transforaminal lumbar interbody fusion; ODI: oswestry disability index; EQ-5D: euro qualitive of life; VAS: visual analog scale; AST: alternative step test; SMT: six-meter walk test; STS: sit-to-stand test; TUG: timed up and go test; SF36: 36 item short form survey; JOABPEQ: Japanese orthopedic association back pain evaluation questionnaire; BMD: bone marrow density; PLIF: posterior lumbar interbody fusion; RDQ: Roland-Morris disability questionnaire; SF36,PCS: 36 item short form survey, physical component summary; JOA score: Japanese orthopedic association score; PCS: pain catastrophizing scale; HADS: hospital anxiety and depression scale; FABQ: fear-avoidance beliefs questionnaire; ASA: American anesthesiologists’ society score; mFI: modified frailty index; SII: surgical invasiveness index; LOS: the length of stay; IQR: interquartile range; OLIF: oblique lateral interbody fusion; PRO: patient-reported outcome; CCI: Charlson comorbidity index; MSII: Mirza surgical invasiveness index; MIS: minimally invasive surgeries; Nil: nothing.

**Table 2:** Diagnostic Tools and Criteria of Sarcopenia in the Included Studies.

Author, Year	Muscle Strength	Muscle Volume	Muscle Function	Diagnostic Algorithm
	Cut-Off Points			
Inoue et al. (2020) [26]	handheld dynamometer	CT: Bilateral psoas muscle and skeletal muscle (at the third lumbar vertebra)	10 m walk test	AWGS: low HGS + low gait speed + low PMI
	①	PMI Male: <6.36 cm^2^/m^2^, Female: <3.92 cm^2^/m^2^	④	
Kwon et al. (2020) [27]	JAMAR plus + hand grip dynamometer	Nil	Alternative step test, 6 m walk test, sit-to-stand test, time up and go test	AWGS: low HGS
	①	Nil	Nil	
Matsuo et al. (2020) [28]	T.K.K.5001 dynamometer	BIA	5 m walk test	AWGS: low HGS + low gait speed + low SMI
	①	②	④	
Sakai et al. (2020) [30]	Jamar-type dynamometer	BIA	10 m walk test	AWGS: low SMI + Low HGS + low gait speed
	①	②	④	
Wada et al. (2019) [33]	T.K.K. 5401 dynamometer	BIA	10 m walk test	AWGS: low SMI
	Nil		Nil	
Toyoda et al. (2019) [32]	T.K.K.5401 dynamometer	BIA	5 m walk test	AWGS: Severe sarcopenia: low SMI + low HGS + low gait speed
	①	②	④	
Charest-Morin et al. (2018) [22]	Nil	CT: total psoas area (L3 transverse process)	Nil	Normalized total psoas area (NTPA)
	Nil	NTPA: lowest quartile	Nil	
Eguchi et al. (2018) [24]	Nil	DEXA	Nil	SMI
	Nil	③	Nil	
Shen et al. (2018) [31]	hand dynamometer	Nil	Nil	AWGS: low HGS
	①	Nil	Nil	
Inose et al. (2018) [25]	Nil	DEXA	Nil	AWGS: low SMI
	Nil	②	Nil	
Eguchi et al. (2017) [23]	Nil	DEXA	Nil	SMI
	Nil	③	Nil	
Park et al. (2016) [29]	dynamometer	BIA	sit-to-stand test, time up and go test	AWGS: low HGS (defined as sarcopenia in our meta-analysis) or low SMI
	①	②	Nil	
Bokshan et al. (2016) [21]	Nil	CT: bilateral psoas muscle at the fourth lumbar transverse process	Nil	TPA (lowest third)
	Nil	TPA: lowest third	NIl	
Zakaria et al. (2015) [34]	Nil	MRI: bilateral psoas muscle area at the fourth lumbar vertebra	Nil	TPA (lowest third)
	Nil	TPA: lowest third	Nil	

AWGS: Asian working group for sarcopenia; PMI: psoas muscle mass index; HGS: hand grip strength; SMI: skeletal muscle mass index; DEXA: dual-energy X-ray absorptiometry; BIA: bioelectrical impedance analysis; GS: gait speed; CT: computed tomography; MRI: magnetic resonance imaging; TPA: total psoas area. Cuff off points: ①Male: < 26kg, Female: <18kg, ②SMI: Male: < 7.0 kg/m2, Female:<5.7 kg/m2, ③SMI:<5.46 kg/m2 (normal data for sarcopenia in Japanese male and female), ④Gait Speed<0.8 m/s.

**Table 3:** Quality Assessment by Using the Newcastle-Ottawa Scale for the Included Studies.

	Representative of Sarcopenia Patients	Selection of Control	Ascertain of Sarcopenia Measurement	Outcome of Interest not Present at Start	Comparability of Cohorts	Assessment of Outcome	Enough Follow-Up Period	Adequacy of Follow Up	Total Point
Inoue et al. (2020) [26]	★	-	★	★	★★	★	-	★	7
Kwon et al. (2020) [27]	★	-	★	★	★★	★	-	★	7
Matsuo et al. (2020) [28]	★	-	★	★	★★	★	-	★	7
Sakai et al. (2020) [30]	★	★	★	★	★★	★	★	★	9
Wada et al. (2019) [33]	★	-	★	★	★★	★	-	★	7
Toyoda et al. (2019) [32]	★	-	★	★	★★	★	-	★	7
Charest-Morin et al. (2018) [22]	★	-	★	★	★★	★	★	★	8
Eguchi et al. (2018) [24]	★	-	★	★	★★	★	★	★	8
Shen et al. (2018) [31]	★	-	★	★	★★	★	★	★	8
Inose et al. (2018) [25]	★	-	★	★	★★	★	★	★	8
Eguchi et al. (2017) [23]	-	-	★	★	★★	★	-	★	6
Park et al. (2016) [29]	★	★	★	★	★★	★	-	★	8
Bokshan et al. (2016) [21]	★	-	★	★	★★	★	★	★	8
Zakaria et al. (2015) [34]	★	-	★	★	★★	★	★	★	8

★—numbers of points earned in each cell.

## Data Availability

The datasets used and/or analysed during the current study are available from the corresponding author on reasonable request: contact Dr. Ke-Vin Chang (kvchang011@gmail.com).

## Protocol Registration

This current meta-analysis was registered on INPLASY (INPLASY2020120123) and is available in full on the inplasy.com (https://inplasy.com/inplasy-2020-12-0123/; doi:10.37766/inplasy2020.12.0123).

## Abbreviations

LDSD	Lumbar degenerative spine diseases
RR	Risk ratio
SMD	Standardized mean difference
AWGS	Asian Working Group for Sarcopenia
EQ-5D	EuroQol-5D

## Appendix A. Strategy of Literature Search

PUBMED:

1.(‘lumbar spinal stenosis’ OR spondylolisthesis OR ‘lumbar degenerative disc disease’ OR ‘low back pain’)

2. (‘sarcopenia’ OR ‘frailty’)

3. 1 and 2

EMBASE:

1. (‘lumbar spinal stenosis’ OR spondylolisthesis OR ‘lumbar degenerative disc disease’ OR ‘low back pain’)

2. (‘sarcopenia’ OR ‘frailty’)

3. 1 and 2.
